# New onset colitis in an adult patient with chronic granulomatous disease treated with hematopoietic stem cell transplantation: a diagnostic dilemma

**DOI:** 10.1186/s13223-018-0243-z

**Published:** 2018-05-08

**Authors:** Kara Robertson, Stephen Couban, Desmond Leddin, Imran Ahmad, Lori Connors

**Affiliations:** 1 Department of Medicine, Dalhousie University, QEII-Bethune Building Room 442, 1276 South Park Street, Halifax, NS B3H 2Y9 Canada; 2 Division of Hematology, Dalhousie University, QEII-Bethune Building Room 430 Bethune Building, 1276 South Park Street, Halifax, NS B3H 2Y9 Canada; 3 Division of Gastroenterology, Dalhousie University, QEII-Victoria Building Room 915, 1276 South Park Street, Halifax, NS B3H 2Y9 Canada; 4 Department of Medicine, Hopital Maisonneuve-Rosemont, Universite de Montreal, 5415 de l’Assomption blvd., Montreal, QC H1T 2M4 Canada; 5 Halifax Allergy and Asthma Associates, 5657 Spring Garden Road Suite 503, Halifax, NS B3J 3R4 Canada

**Keywords:** Chronic granulomatous disease, Colitis, Hematopoietic stem cell transplantation

## Abstract

**Background:**

Chronic granulomatous disease (CGD) is a rare primary immunodeficiency characterized by recurrent life-threatening bacterial and fungal infections, granuloma formation and intestinal disease. This disease is caused by defects in NADPH oxidase, which result in the inability of phagocytes (neutrophils, monocytes and macrophages) to destroy certain microbes. The only established curative therapy for CGD is hematopoietic stem cell transplantation.

**Case presentation:**

A 23-year-old Caucasian male with X-linked chronic granulomatous disease underwent a reduced-intensity conditioning, matched unrelated donor peripheral blood stem cell transplant, after which he was started on tacrolimus and mycophenolate for graft-versus-host disease prophylaxis. Seven months later, he was admitted to hospital for nutritional support secondary to odynophagia and anorexia. Upper endoscopy revealed ulcers in his esophagus, and he was initially treated with acyclovir due to the risk of CMV infection until biopsies came back negative for viral colitis. Following a sigmoidoscopy that showed nonspecific colitis, he was started on mesalamine. Although pathology showed a pattern of widespread inflammatory changes initially suggestive of CGD colitis, a peripheral blood chimerism study showed 100% donor alleles suggesting CGD remission. Since this patient’s colitis was refractory to other immunomodulators, and due to its severity, the patient underwent a partial colectomy 1 year after his HSCT and will likely require the removal of the remaining large bowel.

**Conclusions:**

This case demonstrates a unique presentation of colitis in a post-transplant CGD patient. Since CGD colitis could be excluded due to the patient’s recent successful hematopoietic stem cell transplantation, a broad differential diagnosis is required for determining the etiology of this new-onset colitis in this patient with pre-existing chronic granulomatous disease. This case delineates the need for interdisciplinary care and describes a severe case of colitis after hematopoietic stem cell transplantation.

## Background

Chronic granulomatous disease (CGD) is a rare primary immunodeficiency characterized by recurrent life-threatening bacterial and fungal infections, granuloma formation and intestinal disease [1]. CGD is a recessive genetic disorder caused by a defect in one of five genetic loci coding for NADPH oxidase, a critical enzyme required in the phagocytosis pathway.

Due to the genetic nature of this disease, patients with CGD often present with recurrent infections within the first 2 years of life [2]. These recurrent infections often manifest as axillary, perianal or scalp pyodermas, although liver, spleen or pulmonary abscesses are not uncommon [3–5] with granulomata occasionally seen in other organ systems in the body [6]. Colitis is also often seen in patients with CGD who demonstrate severe gastrointestinal symptoms such as abdominal pain, diarrhea, failure to thrive or microcytic anemia [7].

Treatment options for patients with CGD are often limited to infection prophylaxis with trimethoprim-sulfamethoxazole (Septra) for bacterial infection, itraconazole for fungal infections and immunomodulation with interferon gamma to alleviate the persistently pro-inflammatory state. Acute infections are often treated with empiric antibiotics given the immunocompromised state of patients with CGD and their significantly elevated likelihood of developing sepsis [8]. However, hematopoietic stem cell transplantation (HSCT) remains the only established curative therapy for CGD [9], and this has become the standard of care in the pediatric population.

After HSCT, immunocompetency is usually achieved with complete engraftment. However, the two main complications in a post-HSCT patient are graft-versus-host disease (GvHD) or graft failure **[**10**]**. GvHD may present acutely or chronically, with acute GvHD typically presenting in the first 100 days post-transplant [11]. The typical presentation of acute GvHD is characterized by a triad of cutaneous, hepatic and gastrointestinal manifestations. The cutaneous reaction manifests as a distinct erythematous maculopapular rash which can desquamate in late stages, while the hepatic manifestation usually presents hyperbilirubinemia before fulminant hepatic failure. The gastrointestinal manifestation of GvHD typically presents as diarrhea and anorexia which can become a complete ileus in late stages, mimicking the presentation of colitis [12]. Acute GvHD can occur in up to 30% of allogenic matched post-HSCT patients despite a regimen of immunosuppressive therapies for GvHD prophylaxis. Chronic GvHD typically will manifest within the first 2 years post-HSCT outside of the acute GvHD window, and can present with a non-specific malar rash, sicca, arthritis, bronchiolitis obliterans, or bile duct degeneration and cholestasis. Chronic GvHD will develop in 20–50% of post-HSCT recipients, with unmatched donor or prior acute GvHD reaction as significant risk factors [13].

Late-onset acute GvHD has been described where a patient will develop the classic triad of signs characteristic to acute GvHD outside of the first 100 days post-HSCT [14], or even as an overlap with the manifestations of chronic GvHD [15]. As a result, a patient may develop cutaneous, hepatic or gastrointestinal disturbances on a background of non-specific systemic dysfunction well outside of the acute GvHD window [16]. However, graft failure in a post-transplant CGD patient would manifest as return of CGD with classic signs of CGD, such as recurrent abscesses, granulomas or colitis.

Here we describe the workup, clinical course and response to treatment of a patient with CGD who presents 7 months post-allogenic HSCT with a new onset colitis of unknown etiology, and the diagnostic dilemma to identify the etiology of this patient’s symptoms.

## Case presentation

A 23-year-old Caucasian male with X-linked CGD (gp91^*phox*^ mutation) underwent a reduced-intensity conditioning [17], matched unrelated donor peripheral blood stem cell transplant as curative therapy for his CGD. He was then started on tacrolimus and mycophenolate for GvHD prophylaxis post-HSCT. The transplant was well tolerated, with no documented evidence of acute GvHD, but 7 months later, he was admitted to hospital for nutritional support secondary to odynophagia and anorexia. Upper endoscopy revealed ulcers in his esophagus. He was initially empirically treated with IV acyclovir and biopsies were performed while the patient was on prophylactic doses of immunosuppressive therapy. However, these biopsies came back negative for HSV/CMV and IV acyclovir was discontinued. The pathology report showed a non-specific pattern of widespread inflammatory changes. There was no personal for family history of IBD or GI disease in either the donor or recipient prior to HSCT. Following a sigmoidoscopy showing nonspecific colitis, he was started on mesalamine. To rule out HSCT failure and recurrence of CGD, we performed NBT testing and peripheral blood chimerism studies. NBT testing demonstrated proper neutrophil functioning, and peripheral blood chimerism studies (VNTR) showed 100% donor alleles, showing no evidence of CGD or HSCT failure. It was then thought he might have chronic GvHD, and he was treated with IV steroids. Unfortunately, the patient was refractory to this treatment and subsequently had several episodes of lower GI bleeding.

**Fig. 1:**
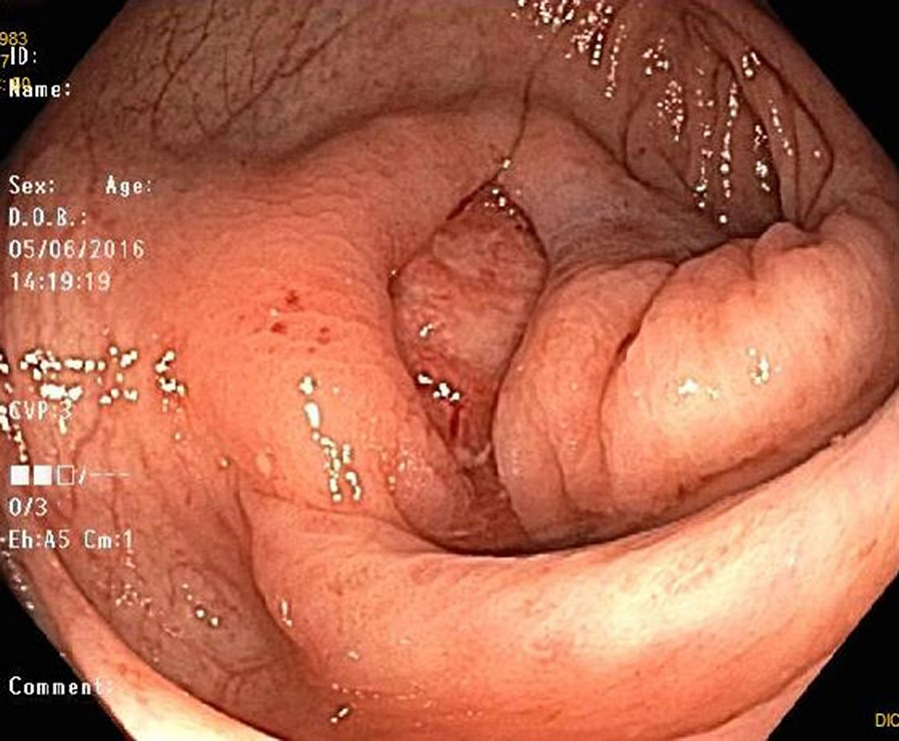
Ulceration visualized on colonoscopy. Colonoscopy conducted 12 months post-HSCT showing healthier tissue in the periphery and a central area of ulceration

**Fig. 2:**
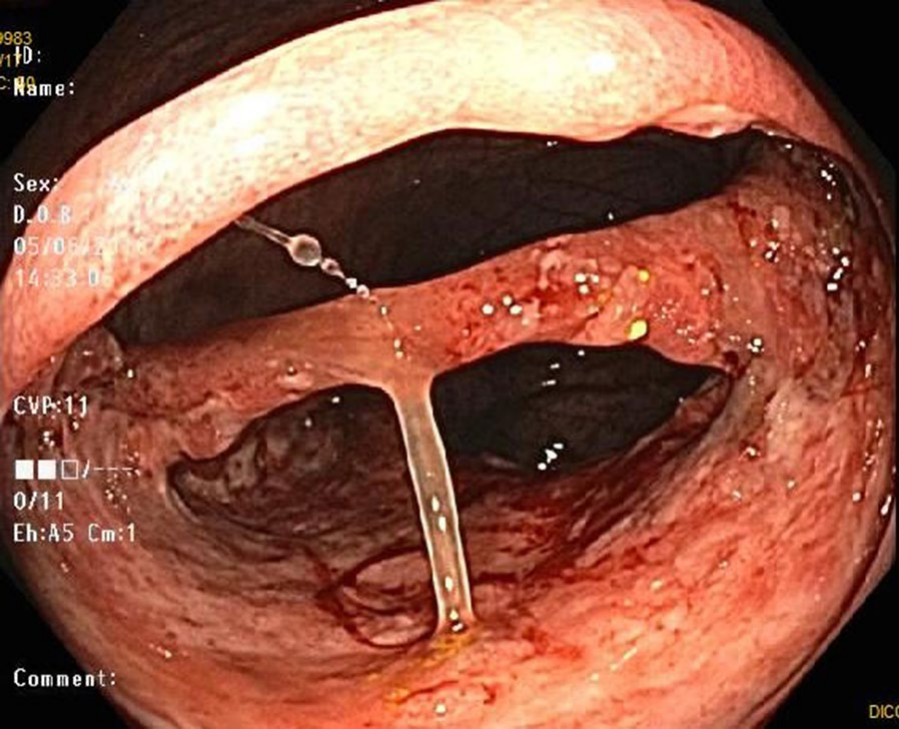
Severe ulceration and mucosal bridging on colonoscopy. Colonoscopy conducted 12 months post-HSCT showing friable tissue and prolific ulceration with 2 mucosal bridges spanning the lumen of the large bowel. Mucus is attached to one bridge of tissue

A colonoscopy demonstrated patchy severe active colitis with ulceration and a solitary granuloma. CMV PCR, acid-fast bacilli and Epstein Barr virus testing was negative. The absolute lymphocyte count was 0.08 × 10^9^ cells/L and flow cytometry revealed CD4—20%, CD8—37%, CD56—63%, CD19—1%, CD45RA—8% and CD45RO—91% (Table 1). He was then started on IVIG at immunomodulatory dosing, his mycophenolate was discontinued, and prednisone and tacrolimus doses were reduced. Repeat colonoscopy 1 month later showed very severe disease with ulceration (Fig. 1) and mucosal bridging (Fig. 2) with no improvement or interval change.

**Table 1:** Lymphocytes by cytokine determinants

Cytokine determinant	Relative abundance (%)
CD4	20
CD8	37
CD56	63
CD19	1
CD45RA	8
CD45RO	91

Flow cytometry on serum lymphocytes shows the relative abundance of CD4+ and CD8+ T cells, CD56+ NK cells, CD19+ B lymphocytes, as well as CD45RA+ naive and CD45RO+ memory T cells

Due to the severity of the colitis, the patient then underwent a partial colectomy. This demonstrated severe patchy chronic active colitis with deep mucosal ulcers and pseudo-inflammatory polyps, with pathology suggestive of Crohn’s disease. The disease in the distal bowel remains active and the patient will likely require removal of the remaining colon.

## Discussion

This patient with chronic granulomatous disease initially presented to us with a new-onset gastrointestinal inflammation and ulceration following HSCT. This was initially suggestive of a diagnosis of CGD colitis, a common gastrointestinal manifestation of X-linked CGD. However, CGD colitis is pathologically distinct from an inflammatory bowel disease colitis due to the presence of hyperpigmented macrophages, a predominantly eosinophilic inflammatory infiltrate, and a scarcity of neutrophils and within the colonic wall [18]. We were therefore able to exclude CGD colitis and HSCT failure with confidence in our patient. After allelic testing, GvHD was then suspected due to the widespread inflammation, and plethora of phagocytes and lymphocytes within the gastrointestinal lining. Although GvHD can manifest with nonspecific symptoms suggestive of upper and lower gastrointestinal tract involvement [19], GvHD serologically presents with marked proliferation of CD4+ and CD8+ T cells, and very few CD56+ NK cells [20], which was not seen on flow cytometry. We were therefore also able to exclude a typical presentation of GvHD from our differential, as the characteristic typical GvHD cutaneous and hepatic reactions were not seen in conjunction with this patient’s gastrointestinal symptoms. However, there were three principle findings that prevent us from ruling out atypical GvHD in our patient. These include presence of apoptotic bodies, mucosal necrosis, ulceration, submucosal fibrosis, and crypt and gland destruction on pathology, as well as detection of alloreactive donor-derived cells against the patient’s gut mucosa, and lastly, our observation of lymphocytic infiltration of the gut mucosa as important yet atypical elements seen in GvHD. As a result, we are not able to rule out an atypical presentation of GvHD for the etiology of this colitis.

In spite of our thinning differential, this post-HSCT patient has presented with esophageal and colonic involvement, and since allelic testing demonstrates entirely donor alleles, the possibility of Crohn’s-like disease secondary to HSCT or atypical GvHD cannot be completely ruled out. The presence of mucosal bridges is a known phenomenon seen in Crohn’s disease [21–24], but pathology only reported nonspecific granulomatous inflammation, not in keeping with the classic histologic findings of Crohn’s disease. Although the risk of IBD is elevated in patients with CGD [25, 26], our gastroenterologist colleagues felt that the absence of autoimmune disease in both the patient’s and donor’s past medical history was not in keeping with an autoimmune inflammatory bowel disease. Verification with the donor registry has confirmed that the donor has not experienced any gastrointestinal complications to date. Regardless, this case would be one of the first documented reports of colitis secondary to HSCT in a patient treated to cure CGD, but the pathology report notes that the possibility of an infectious etiology cannot be excluded, such as an unknown viral complication given this patient’s profound lymphopenia. As yet, we have been unable to determine if this colitis is secondary to IBD, atypical GvHD or an infectious etiology. The vast number of possible etiologies for this colitis presents a challenge in determining the underlying cause of this patient’s illness as well as providing treatment options.

## Conclusion

A broad differential diagnosis is required for colitis presenting after HSCT in a patient with pre-existing CGD. We have been able to rule out common etiologies such as recurrent CGD, typical GvHD, and viral colitis, but we have yet to exclude another Crohn’s-like colitis, an atypical presentation of GvHD or other unknown viral etiology from our differential diagnosis, however the current plan for this patient’s colitis involves future elective total colectomy for disease control, with observation for disease recurrence. This case serves to delineate the importance for multidisciplinary care and describes a severe case of colitis after HSCT.

## Abbreviations

CGD: chronic granulomatous disease
HSCT: hematopoietic stem cell transplant
GvHD: graft versus host disease
NBT: nitroblue tetrazolium
